# Editorial: Modulating Cytokines as Treatment for Autoimmune Diseases and Cancer

**DOI:** 10.3389/fimmu.2020.608636

**Published:** 2020-10-15

**Authors:** Erwan Mortier, Averil Ma, Barbara A. Malynn, Markus F. Neurath

**Affiliations:** ^1^University of Nantes, CNRS, Inserm, CRCINA, Nantes, France; ^2^Department of Medicine, University of California, San Francisco, San Francisco, CA, United States; ^3^University Hospital Erlangen, University Erlangen-Nürnberg, Erlangen, Germany; ^4^Deutsches Zentrum Immuntherapie DZI, Erlangen, Germany

**Keywords:** cytokine, oncology, inflammation, inhibitor, therapy

## Introduction

Cytokines are key mediators in the regulation of the normal immune response. They are ambivalent molecules which can be either beneficial to the treatment of diseases, but can also be harmful and participate in pathogenesis. Indeed, despite regulatory controls at multiple levels, abnormal immune responses involving cytokines can occur and cause various pathologies, including autoimmunity and inflammation-induced cancer. For these reasons, it is crucial to continue efforts focused on understanding the different modes of action of cytokines with an eye toward the design of new selective drugs that modulate cytokine activities that direct beneficial immune responses.

Deregulation of cytokine expression has a complex role in disease pathogenesis and novel therapeutic agents that neutralize cytokines have been successfully translated into clinical practice. For instance, the use of monoclonal anti-TNF antibodies have greatly improved the health of patients suffering from diseases like inflammatory bowel diseases, rheumatoid arthritis, spondyloarthritis, or psoriasis. Furthermore, additional cytokine blockers such as anti-IL-6R antibodies, IL-12/IL-23 p40 inhibitors and IL-23 p19 blockers have been approved for various immune-mediated diseases. The use of effector cytokines (e.g., IL-2, IFNγ) either alone or in combination with other therapeutic reagents, such as checkpoint inhibitors and emerging immunocytokines, is accelerating in cancer immunotherapy. While, IL-2 was approved by the Food and Drug Administration for the treatment of metastatic kidney cancer in 1992 and for metastatic melanoma in 1998, researchers are still working to improve IL-2 efficacy and reduce toxicity. Given the broad range of biological activities of cytokines, the side effects of biologic therapies need to be carefully assessed and warrant the development of new therapeutics with improved specificity of action. Thus, fundamental discoveries on structural features of cytokines in interaction with their different receptor chains could lead to the identification of cytokines with reduced toxicity and increased specificity.

In this Research Topic issue entitled “Modulating Cytokines as Treatment of Autoimmune Diseases and Cancer”, we have compiled 6 original research articles, 1 hypothesis and theory and 6 reviews. This collection is divided into four sections. The first section presents recent knowledge for a better understanding of the mode of action and the structural features of the interaction between cytokines and their receptors. The second section describes new targets for the treatment of autoimmune diseases. The third is devoted to the use of cytokines to induce tolerance and the last section presents different combinations between cytokines and other therapeutic agents for the treatment of cancer.

## Better Understanding of Cytokine Structure/Function and Their Modes of Action

Increasing knowledge of the structural interactions between a cytokine and its receptor chains is fundamental for generating original selective reagents targeting cytokine’s action. In their review, Markovic and Savvides focused on the structure and the mode of signaling assemblies of two closely related cytokines that share the IL-7Rα chain, IL-7 and TSLP. The review of Metcalfe et al. is devoted to the IL-6 family with a focus on IL-11. The authors of both reviews present structural overviews of the two cytokine systems and their involvements in pathological conditions. They also provide an overview of the broad array of potential therapeutic agents in autoimmune diseases to thwart overexpression of the targeted cytokines including monoclonal antibodies, chemical compounds, soluble receptors, and muteins. Along this line, Holgado et al. focused on an original strategy for inhibiting the action of cytokines by generating cytokine-traps. Their approach is based on the generation of molecules consisting of the fusion of receptors chains to form soluble heterodimers capable of capturing cytokines before interacting with their membrane-bound receptors. They are able to efficiently modulate either IL-33 alone by the IL-33-Trap or two cytokines simultaneously using the dual IL-14/13-Trap to inhibit experimental airway inflammation.

Cytokines are primarily described as soluble factors, but they can also be packaged within extracellular vesicles. In their review, Barnes and Somerville provide a new vision of the action of cytokines and lead us to bear in mind that the production of both cytokines and extracellular vesicles play an important role in pathology. These properties could be translated into therapy by engineering extracellular vesicles to deliver immune modulators such as cytokines in pathological conditions.

## Looking for New Targets

The research community is always on the lookout for new targets for the design of novel therapeutics. Thus, it is crucial to deepen our knowledge of relevant targets involved in pathology. Brune et al. focused on IRF5, a key transcriptional regulatory factor of type-I interferon. Hyperactivation of IRF5 has been identified as key factor in several autoimmune diseases, including systemic lupus erythematosus. In their review, Brune et al. provide an original perspective on the complex role of IRF5 and focus on T cell functions and polarization.

Innate lymphoid cells (ILC) are unique cell populations that play important roles in immune defense in response to chronic inflammatory and autoimmune diseases. Schulz-Kuhnt et al. focused on recent knowledge of the functions of human ILCs and provide a comprehensive view of the major regulators, including cytokines, that selectively support the three ILC subpopulations. A better understanding of the regulation of human ILC functions should help researchers use ILCs and modulate their action under inflammatory conditions in the future.

Adipokines are cytokines produced by adipocytes. Among them, visfatin appears to play an important role in the pathogenesis of rheumatoid arthritis (RA) by increasing the adhesion of RA synovial fibroblasts to endothelial cells. Hasseli et al. draw attention to visfatin and other adipokines as potentially interesting targets in the search for RA therapeutics.

In generating new reagents, investigators need to assess their efficacy in relevant animal models. In their study, Lio et al. analyzed the literature that used dry eye disease models to find cells and cytokines that could be targeted in this pathology. They show the involvement of Th1 cells as well as IL-1β and TNFα proinflammatory cytokines. This meta-analysis also prompts precaution when using animal models that do not fully recapitulate human pathology.

## Use of Cytokines to Induce Tolerance

Autoimmune diseases are characterized by the disruption of tolerance to self antigens. Different approaches have been designed by researchers to restore tolerance, including cell therapy by injecting tolerogenic dendritic cells (Tol-DC) or regulatory T (T-reg) cells. Another approach is to target tolerogenic cells directly in vivo. Cauwels and Tavernier proposed an original strategy to expand endogenous Tol-DC in vivo by the administration of AcTakine molecules. The latter consists of a targeting module (VHH is more commonly used) fused to a mutated cytokine with reduced affinity to its cognate receptor. They engineered a Tol-DC AcTaferon with IFN-I to induce tolerance in autoimmune diseases.

IL-2 is an important cytokine for the development of T-reg cells, which constitutively express IL-2Rα. The latter forms a trimeric receptor with IL-2Rβ and the common gamma chain and binds IL-2 with a high affinity allowing T-reg cells to respond to low dose of IL-2. In their study, Ghelani et al. attempt to find the threshold required for IL-2 to selectively expand T-reg cells into effector cells. To this end, they generated a series of IL-2 muteins and found that minimal IL-2 receptor signaling is required to fully expand regulatory T cells and support their immunosuppressive functions.

IL-34 is another cytokine with tolerogenic properties. In their study, Bézie et al. show that CD4 and CD8 FoxP3 regulatory T cells increase significantly when cultured in the presence of monocytes differentiated by IL-34. In addition, human CD8 regulatory T cells grown under these conditions suppress the immune response in a humanized model of acute GVHD by effectively increasing the survival of the graft after organ transplantation by acting on T-regs cells and monocytes. These results demonstrate that IL-34 should also be considered for therapy with regard to its property for promoting the development of regulatory T cells.

## Cytokines in Combination With Other Therapeutic Agents In Cancer

IL-15 is a cytokine that shares with IL-2 an important role in supporting the development and functions of effector cells, such as NK and CD8 T cells. Unlike IL-2, IL-15 does not support regulatory T cells. For these reasons, IL-15 was identified early as a potential candidate for use in cancer immunotherapy. However, IL-15 administrated as monotherapy did not show therapeutic efficacy despite a dramatic expansion of NK and CD8 T cells. In their review, Waldmann et al. present assays that use IL-15 in combination with therapeutic monoclonal antibodies such as anti-CD40, anti-CD20, anti-CD52, anti-EGFR, anti-CTLA-4, anti-PD-1 and anti-PDL-1. Preliminary combination studies show better efficacy than individual agents alone and hold promise for the treatment of patients with metastatic malignancy.

Another way to combine cytokines and therapeutic monoclonal antibodies is to fuse them to generate immunocytokines (ICK). In their study, Shen et al. fused anti-PD-1 antibody to IL-21 muteins. The advantages of such molecules are numerous. They improve both the half-life and bioavailability of cytokines. Targeting cytokine therapies to specific cells may better replicate the paracrine activities of physiologically delivered cytokines. This approach can thus enhance efficacy and limit off-target effects. To restrict IL-21 activity on targeted cells and slow down the clearance of the ICK, the investigators mutated the cytokine to decrease its affinity for its cognate receptor. Preliminary preclinical data reveal that the anti-PD1-attenuated-IL-21 immunocytokine shows promise for anti-cancer indications.

Collectively, the above studies highlight the potential of recombinant cytokines and their inhibitors to more effectively treat autoimmunity and cancer. These results open new avenues for research and may lead to improved therapeutic options in clinical therapy by precision editing of cytokine responses.

## Author Contributions

All authors listed have made a substantial, direct, and intellectual contribution to the work, and approved it for publication.

## Conflict of Interest

The authors declare that the research was conducted in the absence of any commercial or financial relationships that could be construed as a potential conflict of interest.

